# Treatment dismantling pilot study to identify the active ingredients in personalized feedback interventions for hazardous alcohol use: randomized controlled trial

**DOI:** 10.1186/s13722-014-0022-1

**Published:** 2014-12-10

**Authors:** John A Cunningham, Michelle Murphy, Christian S Hendershot

**Affiliations:** National Institute for Mental Health Research, the Australian National University, Canberra, Australia; Centre for Addiction and Mental Health, Toronto, Canada; University of Toronto, Toronto, Canada; Department of Psychiatry, University of Toronto, Toronto, Canada

**Keywords:** Alcohol, Brief intervention, Internet, Randomized controlled trial, Mechanisms of change

## Abstract

**Background:**

There is a considerable body of evidence supporting the effectiveness of personalized feedback interventions for hazardous alcohol use—whether delivered face-to-face, by postal mail, or over the Internet (probably now the primary mode of delivery). The Check Your Drinking Screener (CYD; see www.CheckYourDrinking.net) is one such intervention.

**Objectives:**

The current treatment dismantling study assessed which components of personalized feedback interventions were effective in motivating change in drinking. Specifically, the major objective of this project was to conduct a randomized controlled trial (RCT) comparing the impact of the normative feedback and other personalized feedback components of the CYD intervention in the general population.

**Methods:**

Participants were recruited to take part in an RCT and received either the complete CYD final report, just the normative feedback sections of the CYD, just the personalized feedback components of the CYD, or were assigned to a no-intervention control group. Participants were followed-up at 3 months to assess changes in alcohol consumption.

**Results:**

A total of 741 hazardous drinking participants were recruited for the trial, of which 73 percent provided follow-up data. Analyses using an intent-to-treat approach found some evidence for the impact of the personalized feedback components of the CYD in reducing alcohol consumption on the variables, number of drinks in a week and AUDIT-C (*p* = .028 and .047 respectively; no impact on highest number of drinks on one occasion; *p* = .594). However, there was no significant evidence of the impact of the normative feedback components (all *p* > .3).

**Conclusions:**

Personalized feedback elements alone could provide an active intervention for hazardous drinkers, particularly in situations where normative feedback information was not available.

**Trials registration:**

ClinicalTrials.gov NCT01608763.

**Electronic supplementary material:**

The online version of this article (doi:10.1186/s13722-014-0022-1) contains supplementary material, which is available to authorized users.

Personalized feedback interventions for hazardous alcohol use are now widely used in a variety of modalities [1-3]. These interventions have been applied in both university and general population settings, and in a growing number of countries.

Personalized feedback interventions often have several recognizable components. Most obvious of these are summaries of the amount participants drank, as well as other personalized feedback thought to be relevant, such as amount spent on alcohol, calories consumed, and reports of severity of use according to participants’ scores on a validated scale (e.g., Alcohol Use Disorders Identification Test (AUDIT) [4,5]. Distinct from this personalized feedback is normative feedback—comparisons of how much the person drank (or spent, etc.) to others of a relevant comparison group (e.g., males of the same age group, from the same country; students at the same university). In addition, personalized feedback interventions often also contain sections outlining the dangers of heavy drinking and advice on safe levels of consumption.

Given the evidence base regarding the impact of normative feedback [6], the efficacy of personalized feedback interventions have often been ascribed to this component of the intervention. However, no dismantling trials have been conducted to discern if the normative feedback, the personalized feedback, or a combination of both is the active component of these interventions. Research exploring the active mechanisms of change has been identified as a priority, based on the assumption that understanding the active components of different interventions will allow us to target these interventions more efficiently and increase their effectiveness [7].

The Check Your Drinking intervention (CYD; see www.CheckYourDrinking.net) is a personalized feedback intervention that has its roots in the Drinkers Check-up developed by Miller and colleagues [8]. The CYD has been subjected to four randomized controlled trials in different settings and has fairly consistent evidence of impact on hazardous alcohol consumption [9-12]. The intervention is described in detail elsewhere [13] and contains a variety of personalized feedback elements as well as normative feedback elements. This study tested whether it is the personalized feedback or the normative feedback component of the CYD that motivates change among hazardous drinking participants.

## Methods

Potential participants were recruited via free online classified advertising (Kijiji) in cities across Canada using the following advertisement: “CAMH Internet Study: Drinkers Needed. Research study revising & evaluating Internet-based interventions for alcohol users. Would you like to help? Click here. Compensation provided; not a treatment service.” Interested participants clicked on a link to take them to a more detailed description of the trial. After providing informed consent, participants completed a baseline survey (also online). Primary inclusion criteria were: 1) being 19 years of age or older (legal drinking age in Canada); 2) scoring 8 or more on the Alcohol Use Disorders Identification Test (AUDIT – a score of 8 or more indicates current hazardous drinking) [4,5]; and 3) drinking beyond recommended Canadian safe drinking guidelines (for males, no more than 15 drinks per week and three drinks per drinking day; for females, no more than 10 drinks per week and two drinks per drinking day) [14]. The questionnaire contained a graphic describing a standard drink that in Canada contains 13.6 grams of ethanol.

Participants identified as eligible were sent an email to confirm their interest. Those returning the confirmatory email were randomly assigned to one of four conditions in a 2×2 design to receive: 1) just the normative feedback component of the CYD intervention, 2) just the other personalized feedback information, 3) a no-intervention control condition, or 4) the full CYD intervention (i.e., both the normative feedback and the other personalized feedback components). Those in the no-intervention control condition were not sent any intervention materials but were instead sent a list of the different components of the CYD feedback and asked to think about how useful they would find each of them. See the multimedia appendices associated with this publication for an example of the materials provided in each of the intervention conditions. Participants were sent an email 3 months after being sent their intervention materials that contained a link to the online follow-up survey. Participants who did not respond to the initial email were sent up to two reminder emails. Participants who completed the follow-up survey were emailed an Amazon gift certificate for $10. Example of materials provided in each intervention condition (see Additional files 1, 2, 3 and 4).

### Outcome variables and analysis plan

The outcome variables were: number of drinks in a typical week, largest number of drinks on one occasion, and the AUDIT-C (the consumption subscale of the AUDIT, which includes three items – frequency of drinking, number of drinks per drinking day, and frequency of five or more drinks on one occasion) [15].

Outcome variables were examined for outliers and Winsorized to normalize the distribution by replacing values more than 3 standard deviations from the mean with the next highest value. Analyses were conducted using an intent-to-treat approach. Missing data were replaced with respective baseline values for participants lost to follow-up. In addition, because there were some participants who provided only partial follow-up data, the primary outcome variables were analysed using three separate 2x2 analyses of covariance (respective baseline variable entered as the covariate). The study was approved by the standing ethics committee of the Centre for Addiction and Mental Health.

## Results

A total of 741 participants met eligibility criteria and were randomized to condition. Bivariate analyses were conducted to compare baseline demographics and drinking variables between experimental condition and found no significant differences (*p* > .05). Participants’ mean (SD) age was 29.8 (9.7), 61 percent were female, 59 percent had some post-secondary education, 35 percent were married or living with a partner, 53 percent were full or part-time employed, and half (53%) had a yearly household income of CAN$30,000 or more. Baseline drinking was substantial, with participants reporting an average (SD) AUDIT score of 18.1 (7.4), typical weekly consumption of 24.2 (15.5), and highest number of drinks consumed on one occasion of 14.0 (6.2).The follow-up rate was 73.5 percent (n = 545), and there was no differential loss of participants by experimental condition (*p* > .05).

The analysis of covariance (ANCOVA) for the outcome variable, number of drinks in a typical week, displayed a significant main effect for personalized feedback [F(1,731) = 4.9, *p* = .028] but no significant main effect for normative feedback [F(1,731) = 1.0, *p* = .329] or for the interaction between personalized and normative feedback [F(1,731) = 0.8, *p* = .373]. Post-hoc planned comparisons were conducted to explore the main effect observed. Participants who received the full CYD feedback displayed significantly less drinking at follow-up compared to participants who received no feedback [F(1,360) = 4.6, *p* = .032] and to participants who received just the normative feedback [F(1,360) = 5.8, *p* = .017](baseline values included as a covariate in each post-hoc planned comparison). Participants who received just the personalized feedback component did not display significantly reduced drinking at follow-up compared to participants who received no feedback [F(1,370) = 0.8, *p* = .380] and to participants who received just the normative feedback [F(1,364) = 0.9, *p* = .350]. Table 1 displays the estimated means and standard errors for each of the three ANCOVAs reported here.

**Table 1:** **Estimated Mean** ^**a**^ **(** ***SE*** **) alcohol consumption at 3-month follow-up by intervention condition**

**Variable**	**None (n =187)**	**Normative feedback only (n =183)**	**Personalized feedback only (n =186)**	**Full CYD (n =185)**	
Drinks per week	20.8 (0.70)	20.8 (0.72)	19.9 (0.70)	18.6 (0.71)	P
AUDIT-C^b^	7.0 (0.14)	7.2 (0.14)	6.9 (0.14)	6.8 (0.14)	P
Largest number of drinks on one occasion	11.4 (0.30)	11.5 (0.31)	11.3 (0.30)	11.2 (0.31)	

^a^Baseline values of each variable entered as a covariate into each 2x2 ANCOVA (Normative feedback provided vs. not provided, by personalized feedback provided vs. not provided). P = Main effect of personalized feedback, *p* < .05.

^b^AUDIT-C is a composite measure that consists of respondents’ scores on frequency of drinking, drinks per drinking day, and frequency of five or more drinks on one occasion. Scores range from 0 to 12.

Similarly, the ANCOVA for the outcome variable, AUDIT-C, displayed a significant main effect for personalized feedback [F(1,736) = 4.0, *p* = .047], but no significant main effect for normative feedback [F(1,736) = 0.6, *p* = .444] or for the interaction between personalized and normative feedback [F(1,736) = 0.6, *p* = .428]. Post-hoc planned comparisons found that participants who received the full CYD feedback did not display significantly lower AUDIT-C scores at follow-up, compared to participants who received no feedback [F(1,369) = 0.7, *p* = .413], but did approach a significant reduction compared to those who received just the normative feedback [F(1,365) = 3.8, *p* = .051]. Participants who received just the personalized feedback component did not display significantly reduced drinking at follow-up, compared to participants who received no feedback [F(1,370) = 0.7, *p* = .398], but did display significantly reduced AUDIT-C scores compared to participants who received just the normative feedback [F(1,366) = 4.4, *p* = .036].

Finally, the ANCOVA for the outcome variable, highest number of drinks on one occasion, displayed no significant main effects for personalized feedback [F(1,731) = 0.3, *p* = .594], normative feedback [F(1,731) = 0.001, *p* = .974], or for the interaction between personalized and normative feedback [F(1,731) = 0.03, *p* = .872].

## Discussion

The goal of this research was to identify the active components of a personalized feedback intervention. The CYD is well established and has research supporting its efficacy [9-12]. The CYD also could be divided easily into two components—the personalized feedback component and the normative feedback component. The results of this trial, though weak, would appear to indicate that it is the personalized feedback component that is the active component of the intervention. There were main effects of personalized feedback for two of the three outcome measures – number of drinks in a typical week and the AUDIT-C. However, post-hoc tests exploring these main effects yielded more ambiguous findings, with mixed effects in the cell-by-cell comparisons. These ambiguous findings make it inappropriate to whole-heartedly interpret the results of this trial as clear evidence of the personalized feedback component as the sole active component of the CYD intervention.

An additional challenging element in interpreting the results of this trial is that the impact of the full CYD intervention as compared to those receiving nothing was fairly weak. As an example, our previous trial comparing the CYD to a no-intervention control group found that providing hazardous drinkers access to the CYD resulted in a six-drink-per-week reduction in drinking over and above the reduction observed by participants in the no-intervention control condition at 3-month follow-up [9]. In comparison, the current trial found a two-drink reduction, which, while still statistically significant, is a minimal reduction in alcohol consumption. We speculate that the recruitment method in the current trial resulted in participants who were less concerned about their drinking than they were in receiving an honorarium for completing the trial. Whatever the reason for this small observed impact in the current trial, it does limit the scope within which any impact of the different components of the CYD intervention could display their separate impact.

Given that there is a fairly extensive literature on the impact of personalized norms feedback [6], the fact that there was no clear evidence of the impact of the normative feedback component of the CYD in the current trial was unexpected. Interestingly, a trial of personalized feedback for gambling presented with and without norms also found no impact for the version containing normative information [16]. While one possible interpretation is that the normative feedback is not an active component of the intervention, there are several other factors that lead to difficulties with this conclusion. First, it is challenging to create a pure normative feedback component that does not also include some element of personalized feedback. The appendices included with this paper show examples of the normative feedback-only and the personalized feedback-only interventions. While they ‘hold together’ as an intervention for the reader, the full CYD intervention certainly feels like a more convincing document. This means that the lack of normative feedback component impact observed in the trial could be purely the result of the materials we used, rather than being a generalizable effect. Second, the personalized feedback-only intervention contained more pieces of information (that could act as motivators for change) than the normative feedback-only intervention. A replication of this pilot trial might benefit from starting with the goal of designing separate norms-only and personal feedback-only interventions. The CYD was chosen for convenience and because it had an existing evidence base, but its limitation for the current study is that it was designed as one report, with norms and personalized feedback intermingled. Developing these component interventions anew could also allow for an examination of whether there are higher quality norms and personalized feedback elements evaluated in the extant literature, as the CYD is largely unchanged (baring updating general population norms) since its original version developed in the 1990s [17]. A replication trial would also benefit from concentrating on methods for improving follow-up rates, as the extent of missing data in the current trial makes its interpretation difficult.

One final issue of interest is to consider whether any differences between groups reflect real changes in drinking as opposed to changes in reporting of amount consumed. Our previous work has explored the possibility that normative data could have an impact on amount of drinking reported because of the potential social desirability effects associated with communicating how much others drinks (particularly if that drinking is less than the participants) [18]. This alternate explanation, if true, would argue against the lack of impact of the normative feedback observed in the current trial. However, there is also the possibility that personalized feedback, separate from the presentation of norms, could have a social desirability effect on self-reported alcohol consumption. This alternate explanation of the results cannot be ruled out in the current pilot trial.

Irrespective of these challenges to interpretation of the results in this trial, the fact that the personalized feedback-only intervention showed an independent impact is encouraging from a practical implementation standpoint. That is, normative feedback data is population-specific (whether to the population of a country or of a university). However, Internet interventions are internationally accessible. Demonstrating that a personalized feedback-only intervention can have an impact allows for the possibility that the CYD, or other interventions of this type, can be set up such that, when normative data is not available for a particular participant, a version of feedback without the norms can be provided with the assurance that an active intervention is being offered to the participant.

## Additional files

Additional file 1
**Example of full CYD report.**
Additional file 2
**Example with just normative feedback.**
Additional file 3
**Example with just other personalised feedback.**
Additional file 4
**Materials sent to participants in no intervention condition.**

## Abbreviations

CYD: Check your drinking screener (www.CheckYourDrinking.net)
AUDIT: Alcohol use disorders identification test
AUDIT-C: Alcohol use disorders identification test – consumption items
