# Supplementary material for: Treatment dismantling pilot study to identify the active ingredients in personalized feedback interventions for hazardous alcohol use: randomized controlled trial
Source: Addict Sci Clin Pract. 2014 Dec 10;10(1):1. doi: 10.1186/s13722-014-0022-1 (PMC4288561; doi:10.1186/s13722-014-0022-1)
Supplement: Additional file 4 — Materials sent to participants in no intervention condition. [file 13722_2014_22_MOESM4_ESM.pdf]

MATERIALS SENT TO PARTICIPANTS IN NO INTERVENTION CONDITION

Self-help for Drinkers

Enclosed is a list of sections we are planning to include in a computer program to create a summary of a person's drinking and how it compares to the drinking of other Canadians. Please read it carefully, and think about whether such information would be useful for drinkers.

The Follow-up Questionnaire we send you in 3 months will ask how useful you think each of these sections might be, and about your drinking between now and then.

<u>List of Possible Sections</u>

Graph indicating how your drinking compares to the drinking of others of your age and sex.

Report of the percentage of days in the last year that you drank.

Report of the number of drinks that you drank in the last year.

Report of the estimate of money you spent on drinks in the last year.

Report of the average number of added calories you consumed per drinking day in the last year.

Graph showing your likelihood of having problems as a result of drinking.

Definition of "heavy drinking day".

Recap of areas in which you experienced harmful effects from drinking in the last year.

Your score on the AUDIT questionnaire (which measures whether drinking should be considered problematic) and graph showing where your AUDIT score fits in the range of possible scores.

Explanation of how your body "burns" alcohol, and report of how long it takes you to metabolize 1, 4, and 10 drinks.

Sensible drinking guidelines.
